# Analysis of microgap for root canal filling material filled with a hydrophilic and hydrophobic obturating system

**DOI:** 10.6026/973206300212586

**Published:** 2025-08-31

**Authors:** Subasish Behera, Anushree Tiwari, Naman Vaidya, Soumyaranjan Nanda, Pallavi Mishra, Medha Bhushan, Miral Mehta

**Affiliations:** 1 Department of Conservative Dentistry and Endodontics, Government Dental College and Hospital (SCB), Manglabag, Cuttack, Odisha, India; 2 Independent Researcher, Paoli, Pennsylvania, 19301, USA; 3 Department of Conservative Dentistry and Endodontics, Government Dental College and Hospital, Ahmedabad, Gujarat, India; 4 Private Practitioner, Cuttack, Odisha; 5 Department of Oral and Maxillofacial Pathology, Kalinga Institute of Dental Sciences, KIIT Deemed to be University, Patia, Bhubaneswar, 751024, India; 6 Department of Conservative Dentistry and Endodontics, Modern Dental College, Indore, Madhya Pradesh, India; 7 Department of Pediatric and Preventive Dentistry, Karnavati School of Dentistry, Karnavati University, Gandhinagar, Gujarat, India

**Keywords:** Microgap, root canal obturation, hydrophilic, hydrophobic, resilon, gutta-percha, sealing ability, SEM analysis

## Abstract

The microgap formation associated with hydrophilic and hydrophobic root canal obturation systems is of interest. Hence, thirty extracted human single-rooted teeth were randomly divided into two groups (n=15 each): Group I - obturated (Resilon with Epiphany sealer) and Group II - obturated hydrophobic system (Gutta-percha with AH Plus sealer). After obturation, the samples and microgaps were evaluated using scanning electron microscopy (SEM) at 2000x magnification. Group I (hydrophilic) showed a significantly lower mean microgap of 2.36 ± 0.52 µm, while Group II (hydrophobic) exhibited a higher mean microgap of 5.81 ± 0.89 µm. Thus, hydrophilic obturation systems showed superior sealing ability with minimal microgap formation compared to hydrophobic systems.

## Background:

The primary goal of root canal therapy is to eliminate microbial infection from the root canal system and prevent reinfection by achieving a hermetic seal along the canal walls and at the apex [1]. A critical factor in endodontic success is the quality of the obturation, which depends on the ability of the filling material to adapt closely to the canal dentin and minimize voids or microgaps [2]. Microgaps serve as potential sites for bacterial leakage, which can compromise the long-term prognosis of root canal-treated teeth [3]. Obturating materials can be broadly classified based on their interaction with moisture as either hydrophilic or hydrophobic systems. Hydrophobic materials, such as gutta-percha in combination with resin-based sealers like AH plus, have long been considered the gold standard in endodontics due to their stability and ease of handling [4]. However, they do not chemically bond to dentin and may show limited adaptation in moist canal conditions [5]. Conversely, hydrophilic systems such as Resilon with Epiphany sealer are intended to chemically bind to dentin and thereby the core material, creating a form of so-called Monoblock which can be more seal-tight and cause less microleakage [6]. In laboratory conditions, such materials have shown potential success in sealing capacity and barrier to the invasion of bacteria [7]. Nevertheless, the clinical superiority of hydrophilic systems is questionable because of the diversity of the change in anatomy of canals and the variation in the technique of the operator. Scanning electron microscopy (SEM) is a specific tool to perform the microgap analysis, which has become an accurate way to assess the fit of obturating materials to dentinal walls [8]. Further on, it is assumed that their hydrophilic nature facilitates their penetration into dentinal tubules and the adaptation to canal walls on even in the residual moisture. This feature, which is sometimes hard to eliminate, probably completely during the clinical work, is presumed to be provided by the hydrophilic characteristics of such materials as Resilon and Epiphany [9]. Such an affinity for moisture can decrease the generation of interfacial openings and enhance the long-term seal of the involved obturation. Additionally, the development of a so-called monoblock within the canal, in other words, linking sealer, core material and dentin, is theoretically beneficial as it will stiffen the root structure and prevent the occurrence of vertical root fractures [10]. But the clinical efficacies of these hydrophilic systems are debatable. Although few *in vitro* experiments have reported enhanced sealing capability, other investigators have alleged adoption of hydrolytic degradation with time in these or other structures by destroying their mechanical integrity and the power of their anchors [11]. Conversely, the long-term history of traditional gutta-percha, which is hydrophobic but not a good adhesive, is biocompatible and also dimensionally stable with resin-sealers [12]. Consequently, it is imperative to first identify how these material systems behave in a controlled laboratory environment before coming up with their definitive clinical recommendations. The microgap formation tests present an apt opportunity to measure the sealing ability of obturating agents in a direct manner. SEM offers high-resolution imaging magnification, providing accuracy in evaluating the interface between the filling material and dentinal walls. Earlier research papers have also indicated that there is a considerable inconsistency in microgap formation between different obturation systems, which underpins the necessity of further research [13]. Therefore, it is of interest to evaluate and compare the microgap formation associated with hydrophilic and hydrophobic root canal filling materials using SEM analysis in an *in vitro* setting.

## Materials and Methods:

This *in vitro* study was conducted using thirty freshly extracted, non-carious human mandibular premolars with single straight canals. Teeth with cracks, root resorption, or previous endodontic treatment were excluded. The samples were thoroughly cleaned of soft tissue and calculus and stored in a 0.1% thymol solution until use. All teeth were decoronated using a diamond disc to standardize the root length at 15 mm. Biomechanical preparation of the root canals was carried out using a rotary nickel-titanium system (ProTaper Universal, Dentsply Sirona) up to size F3, ensuring uniformity in canal preparation. Throughout the instrumentation, canals were irrigated with 2.5% sodium hypochlorite and finally flushed with 5 mL of 17% EDTA for 1 minute to remove the smear layer, followed by a final rinse with distilled water. The canals were dried with sterile paper points before obturation. The specimens were randomly divided into two groups (n=15 each). Group I was obturated using a hydrophilic system consisting of Resilon cones and Epiphany SE sealer, while Group II received a hydrophobic system using gutta-percha cones and AH Plus sealer. In both groups, the lateral compaction technique was used for standardization. The access cavities were sealed with temporary restorative material, and all samples were stored at 37°C and 100% humidity for 7 days to allow complete setting of the sealers. Following this period, each specimen was embedded in acrylic resin and sectioned transversely at 3 mm from the apex using a diamond disc under water cooling. The cross-sectional surfaces were polished and examined under a scanning electron microscope (SEM) at 2000x magnification to assess the microgap at the dentin-filling interface. Digital images were captured, and the gap widths were measured at three different points per sample using image analysis software. The mean values for each group were calculated and compared using an independent t-test, with significance set at p < 0.05.

## Results:

The microgap widths observed in both groups were measured and analyzed. Group I (hydrophilic obturation system: Resilon + Epiphany) exhibited comparatively lower mean microgap values than Group II (hydrophobic obturation system: Gutta-percha + AH Plus). The findings are summarized in the following tables. Table 1 presents the mean microgap widths (in µm) observed at three different points (buccal, lingual, and center) for each sample in both groups. Group I showed an average microgap of 2.36 ± 0.52 µm, whereas Group II had an average of 5.81 ± 0.89 µm. A statistically significant difference was noted between the two groups (p < 0.001) (Table 1 - see PDF). Table 2 (see PDF) shows the comparison of microgaps observed at specific locations within each sample. In Group I, the mean microgap was consistently low at buccal (2.22 µm), lingual (2.45 µm), and center (2.41 µm) locations. In contrast, Group II demonstrated larger gaps at all locations (Table 2 - see PDF). Table 3 (see PDF) provides the frequency distribution of samples based on the range of microgap widths. In Group I, 11 out of 15 samples had microgaps less than 2.5 µm, while in Group II, the majority of samples had microgaps exceeding 5.5 µm (Table 3 - see PDF). Finally, Table 4 (see PDF) summarizes the statistical analysis using an independent t-test. The comparison revealed a highly significant difference in mean microgap widths between the two groups (t = 8.72, p < 0.001), confirming that the hydrophilic obturation system provides a better marginal seal (Table 4 - see PDF). These results indicate a superior adaptation of the hydrophilic root canal filling system to canal walls, as evidenced by significantly reduced microgap formation (Tables 1-4 - see PDF).

## Discussion:

The present *in vitro* study assessed and compared the microgap formation of two root canal obturation systems-hydrophilic (Resilon with Epiphany) and hydrophobic (Gutta-percha with AH Plus)-using scanning electron microscopy. The findings demonstrated that the hydrophilic obturation system exhibited significantly smaller microgaps at the dentin-filling interface than the hydrophobic system, suggesting better sealing ability and potentially superior clinical performance. One of the most important goals in root canal obturation is to achieve apical and coronal tightness to avoid microleakage into the coronal flanks and avoid an influx of microorganisms, which is the most common cause of endodontic therapy failure [1, 2]. The very common, biocompatible gutta-percha, being easily placed and having biocompatible properties, does not stick to dentinal walls and depends significantly on the sealing properties of the sealer [3]. AH Plus, which is among the most frequently applied epoxy resin-based sealants, offers satisfactory dimensional stability and solubility as well as poor chemical bonding to dentin and gutta-percha [4, 5]. This restriction could be the reason why bigger microgaps were detected in this study at the hydrophobic group. Resilon, on the other hand, has a thermoplastic composition based on synthetic polyester and is used with Epiphany sealer, a two-cure resin. This system can bond with the walls of the canal and core building material to a single block-like structure that reduces microleakage [6, 7]. The sealer is hydrophilic, thus flowing easily and penetrating dentinal tubules, which improves the seal despite the remaining moisture [8]. As mentioned in several studies, Resilon/Epiphany systems exhibit, when compared to gutta-percha/AH Plus, less microleakage [9, 10], and this is also indicated in the present findings. SEM evaluation has been a reliable tool for assessing the integrity of root canal obturation due to its high magnification and resolution capabilities [11]. The reduced microgaps in Group I, as seen in SEM analysis, reflect the superior adaptation of the hydrophilic system, which could translate into improved resistance against bacterial penetration. This is particularly significant as studies have shown that even minor interfacial gaps can harbor bacteria and biofilms, ultimately leading to periapical pathosis [12, 13]. Despite its advantages, the Resilon/Epiphany system has certain drawbacks. Some researchers have raised concerns regarding its long-term durability and hydrolytic stability, as degradation over time may weaken the sealer-dentin bond [14]. Additionally, clinical studies comparing long-term success rates between Resilon and gutta-percha have shown mixed outcomes, indicating that *in vitro* advantages do not always equate to clinical superiority [15, 16-17]. Nevertheless, the findings of this study support the hypothesis that hydrophilic systems may offer enhanced sealing capability through better adaptation to the canal walls. The clinical implication is that such systems could be especially beneficial in cases where canal dryness is difficult to achieve or where added reinforcement of the root structure is desired. Future research should include long-term evaluations of leakage resistance, bonding durability, and clinical outcomes through randomized controlled trials. Additionally, assessment under thermocycling and mechanical loading could further validate the sealing behavior of these materials under functional stresses that mimic oral conditions.

## Conclusion:

The hydrophilic obturation system (Resilon with Epiphany) showed significantly better adaptation to canal walls and reduced microgap formation compared to the hydrophobic system (Gutta-percha with AH Plus). Thus, we show that hydrophilic materials may offer improved sealing potential, which could enhance the long-term success of root canal therapy.
